# PReS-FINAL-2011: Preliminary validation of a new hybrid measure of muscle strength for juvenile dermatomyositis

**DOI:** 10.1186/1546-0096-11-S2-P24

**Published:** 2013-12-05

**Authors:** D Marafon, A Consolaro, C Ferrari, S Dalprà, A Madeo, A Providenti, C Malattia, N Ruperto, A Civino, A Martini, A Ravelli

**Affiliations:** 1 Istituto Giannina Gaslini, Genova, Italy; 2 Azienda Ospedaliera Card. G. Panico, Tricase (LE), Italy

## Introduction

Juvenile dermatomyositis (JDM) is a multisystem vasculopathic disease characterized by muscle inflammation that causes symmetrical muscle weakness. Assessment of muscle strength is, therefore, a fundamental component of the clinical evaluation of children with JDM. This assessment is traditionally made using the 8-muscle Manual Muscle Testing (MMT) and the Childhood Myositis Assessment Scale (CMAS). However, the MMT does not cover all muscles or muscle groups affected in JDM, namely abdominal muscles. Although the CMAS is more comprehensive than the MMT, it is lengthy and, therefore, may not be feasible in a busy clinical setting or when a physical therapist is not available.

## Objectives

The study aim was to investigate the construct validity of a new hybrid measure of muscle strength, named hybrid MMT/CMAS (hmc), developed by merging the MMT with 3 items of the CMAS.

## Methods

The hmc is composed of all 8 items of the MMT and the following items of the CMAS: 1) head lift; 2) sits-ups; 3) floor rise. Item 3) is recoded on 0 to 9 scale. The total score of the hmc ranges from 0 (worst) to 100 (normal). The hmc has been validated over two groups of patients: 1) 322 children with JDM enrolled in a multinational long-term outcome survey (Ravelli et al. AC&R 2010;62:63-72); 2) 294 children with DMG included in a multinational study validation of the preliminary definition of clinical response led by PRINTO (Ruperto N. AC&R 2008). Validation procedures were conducted by comparing the correlation of the hmc, MMT and CMAS with other conventional measures of JDM activity, physical function and damage. Correlations were computed by means of the Spearman's correlation coefficient and were considered good, moderate, or poor when the r_s _was > 0.7, 0.4-0.7, or < 0.4, respectively. The ability to catch the change over time was assessed by calculating the standardized response mean (SRM) between 2 consecutive visits (the SRM was considered satisfying if> 0.80).

## Results

The Spearman's correlations of hmc, MMT and CMAS with other measures of disease activity and damage are presented in the table 1.

**Table 1:** 

	MYOACT Glob VAS	Parent Global	DAS	MITAX	MYOACT Muscle VAS	CK	CHAQ	MDI Glob VAS	MDI Muscle VAS	MDI Extent	MDI Severity
Hmc	-0.54	-0.35	-0.58	-0.47	-0.68	-0.23	-0.64	-0.46	-0.52	-0.47	-0.44

MMT	-0.58	-0.35	-0.58	-0.47	-0.69	-0.21	-0.62	-0.47	-0.54	-0.46	-0.43

CMAS	-0.42	-0.28	-0.50	-0.40	-0.61	-0.27	-0.54	-0.40	-0.47	-0.43	-0.39

The SRM HMC, MMT, CMAS was, respectively, 0.90, 0.80 and 0.89.

## Conclusion

We have developed a new hybrid measure of muscle strength in JDM, which is more comprehensive than the MMT and more feasible than the CMAS. Overall, the construct validity of the hmc was superior to that of the MMT and CMAS.

## Disclosure of interest

None declared.

